# Labeling adipose derived stem cell sheet by ultrasmall super-paramagnetic Fe_3_O_4_ nanoparticles and magnetic resonance tracking *in vivo*

**DOI:** 10.1038/srep42793

**Published:** 2017-02-21

**Authors:** Shukui Zhou, Ting Yin, Qingsong Zou, Kaile Zhang, Guo Gao, Joseph G. Shapter, Peng Huang, Qiang Fu

**Affiliations:** 1 Department of Urology, Affiliated Sixth People’s Hospital, Shanghai Jiao Tong University, Shanghai, China; 2 Institute of Nano Biomedicine and Engineering, Shanghai Engineering Research Center for Intelligent Diagnosis and Treatment Instrument, Department of Instrument Science and Technology, School of Electronic Information and Electrical Engineering, Shanghai Jiao Tong University, Shanghai, China; 3 School of Chemical and Physical Sciences, Flinders University, Bedford Park, Adelaide 5042, Australia

## Abstract

Cell sheet therapy has emerged as a potential therapeutic option for reparation and reconstruction of damaged tissues and organs. However, an effective means to assess the fate and distribution of transplanted cell sheets in a serial and noninvasive manner is still lacking. To investigate the feasibility of tracking Adipose derived stem cells (ADSCs) sheet *in vivo* using ultrasmall super-paramagnetic Fe_3_O_4_ nanoparticles (USPIO), canine ADSCs were cultured and incubated with USPIO and 0.75 μg/ml Poly-L-Lysine (PLL) for 12 h. Labeling efficiency, cell viability, apoptotic cell rate were assessed to screen the optimum concentrations of USPIO for best labeling ADSCs. The results showed ADSCs were labeled by USPIO at an iron dose of 50 μg/ml for a 12 h incubation time, which can most efficiently mark cells and did not impair the cell survival, self-renewal, and proliferation capacity. USPIO-labeled ADSCs sheets can be easily and clearly detected *in vivo* and have persisted for at least 12 weeks. Our experiment confirmed USPIO was feasible for *in vivo* labeling of the ADSCs sheets with the optimal concentration of 50 μg Fe/ml and the tracing time is no less than 12 weeks.

Cell sheet technology has been widely applied in the field of regenerative medicine and tissue engineering for the past few years. In the absence of a biomaterial scaffold, it requires the non-enzymatic harvesting of cultured cells and creates a contiguous sheeting structure with extracellular matrix (ECM) and intact cell-cell junctions 1,2,3. Because they are highly bioactive and can be easily handled and manipulated, cell sheets can be used to build 3D soft tissues or organs and avoid the defects such as significant cell loss due to trypsinization and difficulty controlling the location of the transplanted cells caused by direct cell injection. The time and thickness of cell sheet formation are closely related to the capability of cell proliferation and cell type.

Adipose-derived stem cells (ADSCs) are one of the most common stem cell types to be applied in autoplastic transplantation. Compared with other mesenchymal stem cell types isolated from cartilage and bone marrow, ADSCs possess the highest proliferation potential and exhibit high tolerance to serum deprivation-induced cell apoptosis^4^. Adipose tissue contains a high content of ADSCs and quantities of 0.7 × 10^6^ ADSCs can be obtained per gram of adipose tissue^5^. Furthermore, adipose tissue is abundant in body and there is no effect on the body function after removing a small amount of fatty tissue. Recently, ADSCs sheet transplantation has shown the potential to be used for repair and reconstruction of damaged tissues and organs, including myocardial infarction^6^,^7^, diabetic ulcers^8^ and full-thickness defect wound healing^9^. However, an effective means to assess the fate and distribution of transplanted cell sheets in a serial and noninvasive manner is still lacking.

To track cell sheet survival and migration *in vivo*, it is important to be able to provide serial images from weeks to months after transplantation. Magnetic Resonance Imaging (MRI) offers an established and high-resolution approach to dynamically and persistently detect a small fraction of labeled cells *in vitro* and vivo. Thus it can be used as an ideal tracer method. At present, there are two main groups of paramagnetic contrast agents used for MRI, gadolinium (Gd) based chelates and iron oxide (Fe) based particles. Gadolinium rhodamine dextran (GRID) is the most commonly used MR contrast agents in clinical practice. However, GRID significantly increases the level of reactive oxygen species (ROS) and affects cell proliferation^10^. Iron is a basic element in cellular metabolism, and involved in a series of crucial physiological events, such as oxygen transport, mitochondrial respiration, and DNA synthesis^11^. Many studies have shown labeling with optimized superparamagnetic iron oxide nanoparticles (SPIO) does not trigger cell apoptosis, and does not impair cell survival or proliferation capacity^12^,^13^,^14^,^15^. SPIOs are divided into three main categories according to different hydrodynamic diameters, including oral SPIO, standard SPIO, and ultrasmall SPIO (USPIO). For USPIO, the hydrodynamic diameter size of nanoparticle is less than 50 nm^16^. MR signal enhancement is closely associated with particle size, and the smaller iron oxide provided greater signal enhancement and prolonged signal enhancement^17^. From early reports, USPIO has been evaluated as an MR contrast agent for imaging cells and scaffolds *in vitro* and *in vivo*^12^,^18^,^19^,^20^. Nevertheless, there has been no report on USPIO-labeled cell sheets and their use to further obtain non-invasive imaging information on transplanted USPIO-labeled cell sheet *in vivo*. In this study, we chose ADSCs as seed cells and describe a simple protocol to label ADSCs sheets using USPIO at optimized concentration and demonstrate the feasibility of noninvasive imaging of labeled ADSCs sheet after transplantation *in vivo*.

## Experimental Procedures

### Materials

Ferric citrate, ferrous (II) sulfate heptahydrate (FeSO_4_.7H_2_O, 99%) and NaOH were purchased from by Sinopharm Chemical Reagent Co., Ltd. (Shanghai, China). Ascorbic acid (AA) was obtained from Aladdin Chemical Reagent Co., Ltd. (Shanghai, China). The Cell Counting Kit-8 assay (CCK-8) was purchased from Dojindo Laboratories (Japan). Cell culture products and reagents were purchased from Thermo Scientific (Rockford, IL, USA). Beagle dogs, at the start of the study 10 months of age, and nude mice of 4 weeks of age were provided by the Shanghai sixth people’s hospital Animal Laboratory. The experimental protocol was reviewed and approved by the Ethics Committee and is in compliance with the guidelines for the ethical treatment of animals made by the International Council for Laboratory Animal Science. The water used in all experiments was ultrapure water, which was purified by a Milli-Q Water System (18.2 MΩ cm, Millipore Co., USA). The life sciences committees of *Shanghai Jiao Tong University* approved the experiments, and all experimental procedures were in agreement with institutional use and care regulations.

### Synthesis and characterization of USPIO

Continuing from our previous studies^21^,^22^, herein we developed a hydrothermal method for controllable synthesis of USPIO nanoparticles. The USPIO nanoparticles were prepared by a hydrothermal method using FeSO_4_·7H_2_O, ferric citrate and ascorbic acid as raw materials. In brief, 10 mL FeSO_4_·7H_2_O solution was added to a 30 mL ferric citrate solution in a molar ratio of 2:1 under strong stirring at room temperature. 0.6 mmol ascorbic acid as antioxidant was dissolved in the mixture, and then the pH of the solution was brought to 10 using a 1.5 M NaOH solution. Subsequently, the obtained precursors were poured into a 50 mL Teflon-lined autoclave, which was kept at 200 °C for 10 h and then returned to ambient temperature. The resulting solution was dialyzed by MWCO 14 kDa of dialysis bag for 24 h. To remove bacteria, the above Fe_3_O_4_ nanoparticle solution was then filtered through a 0.22 μm nylon filter. The crystallinity of the synthesized USPIO was determined with an X-ray powder diffractometer (XRD, Rigaku, Japan) using Cu Kα radiation at 1.5418 Å at a scanning rate of 5° min^−1^. Zeta potential measrements were carried out using a NICOMP 380 ZLS ζ potential/particle sizer (PSS Nicomp, USA). Transmission electron microscopy (FEI Tecnai G2 Spirit Twin, Czech Republic) was used to observe the crystal structure and sizes.

### Cell cultures

As a common large experimental animal, canines were used in our study for abundant subcutaneous fat, their similarity to human physiology and strong disease resistance. The primary culture of Canine ADSCs were prepared as reported previously^23^. Briefly, ADSCs were isolated by an enzymatic digestion method. After general anesthesia, primary ADSCs were obtained from fat tissue in the groin of beagle dogs, then digested with 0.1% collagenase I for 1 h at 37 °C. The digested cells were collected after filtration through a cell strainer. The primary ADSCs were maintained in low glucose Dulbecco modified Eagles (DMEM, Gibco, NY, USA) supplemented with 10% fetal bovine serum (FBS, Gibco, NY, USA), 100 U/mL penicillin (Gibco, NY, USA), 100 mg/mL streptomycin sulfate (Gibco, NY, USA). The culture medium was replaced every 2–3 days.

### Cell sheets preparation and labeling

Canine ADSCs at passages 2 were grown in 12-well plate until cell growth reached 80–90% confluence. The polyamine poly-L-lysine (PLL) hydrobromide (Sigma, St Louis, Mo, 175,000 MW) was used as the transfection agent. Canine ADSCs were incubated with USPIO and 0.75 μg/ml PLL. These culture media containing the USPIO-PLL complex were added to the ADSCs such that the final concentration of Fe in USPIO was 5 μg/mL, 10 μg/mL, 25 μg/mL, 50 μg/mL, 100 μg/mL, respectively. Meanwhile, unlabeled ADSCs were used as control. The cell cultures were incubated for 12 h at 37 °C in 5% CO_2_ atmosphere. After incubation for 12 h, cells were washed three times with phosphate-buffered saline (PBS) and further cultured in low glucose DMEM supplemented with 10% FBS. For cell sheet labeling, ADSCs at 5 × 10^4^ per cm^2^ were seeded in a 60 mm Temperature-Responsive Cell Culture Surface (Thermo Fisher Scientific, San Jose, CA, USA) until the cell growth reached 90%-100% confluence (2–3 days). Then, the optimum concentration of USPIO was used to label the ADSCs. Vitamin C catalyzes the hydroxylation of prolyl and lysyl residues in the collagen polypeptide chains, allowing interaction of the collagen subunits, adding structural stability to the collagen fibers, thus it plays a vital role in the production of collagen^24^. In this study, the secretion of extracellular matrix were stimulated with 100 μg/mL vitamin C (Sigma, St.Louis, USA) for 3 days, and then 50 μg/mL vitamin C (Sigma, St Louis, USA) for 18 days to form the cell sheet. The culture medium was replaced every 2 days.

### Prussian blue staining and Iron Quantificationh

After 12 h incubation with varying concentrations of USPIO, cells were washed three times with PBS and intracellular iron oxide distribution was detected by Perl’s Prussian blue staining kit (Solarbio science & Technology, Beijing, China). Cells were fixed for 15 minutes in 4% paraformaldehyde, and then incubated for 25–30 minutes with 10% potassium ferrocyanide, rewashed twice with PBS, and counterstained with nuclear fast red for 10 minutes. Cells containing intracytoplasmic blue granules were defined to be Prussian blue staining positive (PB-positive). Cells labeled with varying concentrations of USPIO were trypsinized and washed 5 times with PBS, then detached, counted and resuspended in a 37% HCl solution. The iron content per cell was obtained by inductively coupled plasma mass spectroscopy (ICP-MS, MICI, Suisse). Three replicates at each concentration were measured for statistical analysis.

### Cellular cytotoxicity assessment

CCK-8 is a sensitive non-radioactive colorimetric assay for detecting the number of viable cells in cytotoxicity and cell proliferation assays^25^. The sensitivity of cell proliferation assays using CCK-8 is higher than other tetrazolium salts, such as XTT, MTT, WST-1 or MTS^25^. According to the manufacturer’s instructions, cellular cytotoxicity of different concentrations of USPIO-labeled ADSCs was determined with a cell counting Kit-8 (Dojindo, Tokyo, Japan). 100 μL ADSCs suspensions (2 × 10^3^/well) were added to 96-well plates. The cells were cultured to 80–90% confluence, different concentrations of USPIO and 0.75 μg/ml PLL was added to each well for 12 hours incubation. After washing three times with PBS, ADSCs were continuously cultured in 100 μL low glucose DMEM supplemented with 10% FBS. 10 μL of CCK-8 was added in 96 wells every 24 hours. After 2 h incubation, optical density (OD) values at 450 nm were measured with a standard microplate reader (TECAN, Männedorf, Switzerland). This experiment was continued for 7 days, then growth curves were created to compare varying concentrations of USPIO-labeled and unlabeled cells.

### Cellular apoptosis

Varying concentrations of USPIO and 0.75 μg/ml PLL were added to the cultures for 12 hours. According to the manufacturer’s instructions, apoptotic cells rate were assessed by an Annexin V-FITC/PI detection kit (BD Biosciences; San Diego, CA, USA). Briefly, the cells were collected by centrifugation at 500× g for 3 min and washed three times with cold PBS. Approximately 1 × 10^6^ cells were resuspended in 250 μL of manufacturer-supplied 1 × binding buffer, and USPIO-labeled and unlabeled ADSCs were incubated with 2 μL of annexin V-FITC and 10 μL of propidium iodide (PI). After 30 minutes incubation in the dark at 25 °C, the cells were then washed twice with annexin medium and analyzed with flow cytometer (Beckman Coulter, Miami, FL, USA).

### Transmission electron microscope

Transmission electron microscopy (FEI Tecnai G2 Spirit Twin, Czech Republic, operating at 80 kV) was used to analyse the intracellular distribution of USPIO in USPIO-labeled cells. First, the optimal concentration of USPIO that give the high transfection efficiency without any effects on cell growth was screened *via* the above steps. Then, the optimal concentration of USPIO-labeled and unlabeled ADSCs was fixed in 2.5% glutaraldehyde for 4 h, post-fixed in 1% osmium tetroxide in the same buffering solution for 2–3 h, contrasted in block with 0.5% uranyl acetate for 2 h, dehydrated using graded ethanol/acetone and included in Araldite^®^ resin. The ultra-thin cuts (50–60 nm) were contrasted in alcoholic solution saturated with uranyl acetate and lead citrate for 20 minutes, then observed and analyzed in FEI Tecnai G2 Spirit Twin.

### Multipotent differentiation of USPIO-labeled ADSCs

To observe the differentiation potential of USPIO-labeled ADSCs, osteoblast, myoblast and adipocyte differentiation assays were performed *in vitro*. The unlabeled ADSCs were used as a control group. Osteogenic differentiation of ADSCs was cultured under the osteogenic induction medium for 3 weeks, containing 90% low glucose DMEM supplemented with 10% FBS, dexamethasone, 50 μg/ml ascorbic acid and b-glycerol phosphate. For adipogenic differentiation, ADSCs were incubated in adipogenic medium for 3 weeks, which consists of 90% DMEM supplemented with 10% FBS, insulin, indomethacin, 1-methyl-3-isobutylxanthine, and dexamethasone. Osteogenic and adipogenic differentiation were detected by alizarin red staining and oil red staining in accordance with manufacturer’s instructions, respectively. In a previous study, our group isolated ADSCs and induced them into myoblast with 5-Azacytidine *in vitro*, and successfully treated stress urinary incontinence of rat^26^. Similarly, we also induced the myogenic differentiation of USPIO-labeled and unlabeled ADSCs by 5-Azacytidine for 3 weeks. The muscle protein expression of Desmin (1:300 antibody dilution, SantaCruz Inc, Santa Cruz, CA) was detected by indirect immunofluorescence staining and observed under the fluorescence microscope.

### Transplantation of USPIO-labeled ADSC sheet

For easy operation and elimination of immune rejection, immune-deficient nude mice were used as experimental animal for *in vivo* transplantation further assessed the fate of the USPIO-labeled cells in the transplanted areas by MRI. After 12 h incubation at the optimal concentration of USPIO-containing medium, ADSCs were washed three times with PBS, and then continuously cultured for 21 days and formed cell sheets with the stimulation of vitamin C. The culture medium was replaced every 2 days. As a flexible and thin sheet tissue composed of cells and ECM, the cell sheet is hard to transplant smoothly under the skin. After cell sheet formation, USPIO-labeled and unlabeled ADSC sheets were folded and stacked to cell sheet “pellets” with cell scraper, which made it easier to transport the sheets and fix them *in vivo*. Before transplantation of the cell sheet “pellets”, nude mice (4 weeks) were anesthetized with pentobarbital-sodium (25–30 mg/kg ip). A 1.5 cm incision was made in the dorsal skin, and underlying space between skin and muscle was exposed. Then, three cell sheet “pellets” from each group were transplanted onto the under skin space and the incision was closed with 5–0 nylon sutures.

### Magnetic resonance imaging *in vitro*

Varying concentrations of USPIO (5 μg Fe/mL, 10 μg Fe/mL, 25 μg Fe/mL, 50 μg Fe/mL, 100 μg Fe/mL) labeled and unlabeled cells were washed twice with PBS, trypsinized, fixed through incubation with 4% paraformaldehyde for 15 minutes and cells collected at 500× g centrifugation for 3 min. Then, 2 × 10^5^ cells were resuspended with 1 mL PBS in 1.5 mL tubes. MR imaging (3.0 T; PharmaScan, Bruker, Germany) was used to determine the sensitivity of MRI for the detection of varying concentrations of USPIO-labeled cells using multi-slice T2- weighted spin echo sequences. Imaging parameters for T2-weighted turbo spin-echo (SET2WI) were as follows: repetition time (TR) = 470.1 ms, echo time (TE) = 7.5 ms, field of view (FOV) = 2.5 × 2.5 cm^2^, matrix = 384 × 384, slice thickness = 0.2 mm, spacing layer = 0.45 mm, and flip angle = 15°. An equal amount of PBS in 1.5 mL tubes was used as control.

### Magnetic resonance imaging *in vivo*

To further observe the magnetic resonance images of transplanted USPIO-labeled ADSCs sheet, 50 μg Fe/mL USPIO-labeled and unlabeled ADSCs sheet “pellets” were examined by MRI 1 week, 4 weeks and 12 weeks after transplanting the cell sheets. To perform the MRI, animals were anesthetized with pentobarbital-sodium (25–30 mg/kg ip). After a gradient-echo localizer in three spatial directions, the T2-weighted MR images were acquired using Rapid Acquisition with Relaxation Enhancement sequence, specific parameter as follows: TE = 71 ms, TR = 3500 ms, RARE factor = 8, number of averages = 8, slice thickness = 1 mm, FoV = 70 × 100 mm, matrix = 384 × 384.

### Histopathology

In week 1, 4, 12 after labeling and embedding into nude mice, nude mice were sacrificed and transplanted USPIO-labeled and unlabeled ADSC sheet “pellets” were removed and further fixed by 4% paraformaldehyde for 24 h, followed by paraffin embedding and sectioning (5 μm in thickness). The sections were HE-stained for microscopic examination. Meanwhile, the presence of iron oxide in the transplanted area was detected by Prussian blue staining. The specific procedures of Prussian blue staining are described in the Prussian blue staining and Iron Quantification section. Finally, the differentiation of ADSC sheets *in vivo* was investigated 12 weeks after transplantation. Alizarin red staining (paraffin section), oil red staining (frozen section) and immunohistochemical staining (Desmin, 1:300 antibody dilution, paraffin section) were used to analyze the osteogenic, adipogenic and myogenic differentiation, respectively.

### Statistical analysis

All data are expressed as means ± SD. To identify significant differences in the labeling efficiency, cellular apoptosis rate and grey value of MR imaging, one-way analysis of variance and unpaired student t test was applied. Cellular cytotoxicity of varying concentration of USPIO-labeled and unlabeled groups were analyzed with repeated measurement analysis of variance. P values less than 0.05 were identified to be statistically significant differences.

## Results and Discussion

**Figure 1:**
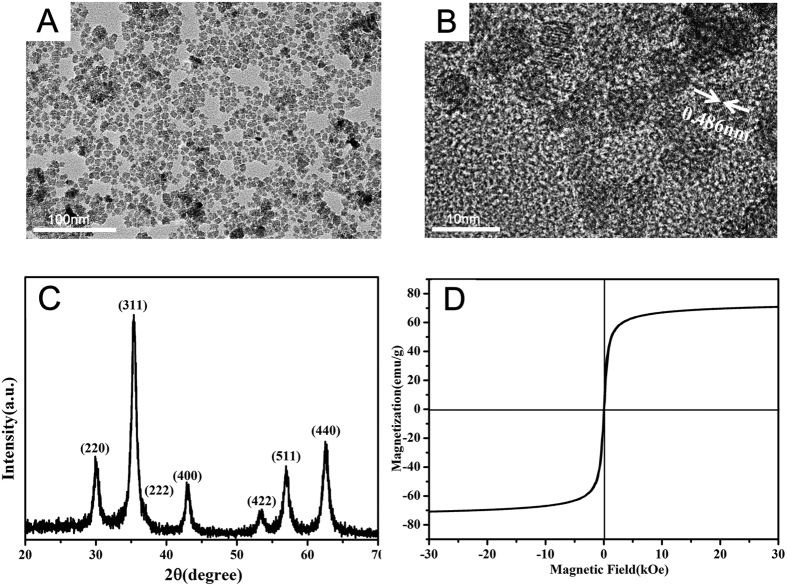
Characterization of the as-prepared USPIO. (**A**) TEM image, scale bar: 100 nm. (**B**) HRTEM image of USPIO, scale bar: 10 nm. (**C**) XRD patterns of the as-prepared USPIO, (**D**) magnetization curves measured at 300 K (**D**).

Flow cytometry analysis indicated, the primary cultured ADSCs in our study were negative for hematopoietic markers CD34 and CD45, and were strongly positive for MSC-related markers CD44, CD90 and CD105, which confirmed the stem cell origin of ADSCs (Supplementary Data 1). Transmission electron microscopy (TEM) images were used to observe the crystal structure and sizes. TEM images showed the monodisperse USPIO nanoparticles were approximately spherical with an average diameter of 10 ± 2 nm (Fig. 1A). High-resolution transmission electron microscopy (HRTEM) of a representative USPIO was observed in Fig. 1B. The lattice distance was measured to be 0.486 nm, consistent with the standard cubic Fe_3_O_4_ structure^27^. The USPIO were further characterized by the X-ray diffraction (XRD) (Fig. 1C). The XRD diffraction peaks of the as-synthesized USPIO were also shown to be due to the cubic Fe_3_O_4_ structure (JCPDS no.19–0629). The excellent saturation moment of the USPIO nanoparticles was ~71 emu g^−1^, indicating that the synthesized USPIO are superparamagnetic, which facilitate the magnetic resonance imaging of medical diagnosis. The hydrodynamic size of nanoparticles in water was approximately 10.5 ± 5.169 nm (PDI = 0.122), which was slightly bigger than the size in TEM. It may be attributed to the hydration of the ligand on the surface of USPIO in ultrapure water, in accordance with the published literature^28^.

USPIO nanoparticles have attracted considerable interest due to their biocompatibility, biodegradability and excellent magnetic properties^29^. An ideal tracer agent requires high detection sensitivity while not having an influence on the activity of cells. There is considerable differences in the doses of USPIO reported in literature. Chen *et al*.^14^ showed labeling endothelial are progenitor cells with USPIO at a concentration of 28 μg Fe/mL for 24 h was nearly 100% efficient and did not affect cell viability. Paik *et al*.^30^ showed that iron nanoparticles used at a concentration of 1% were non-toxic to the bone marrow cells and both the secretion of cytokines and expression of cell surface markers will not be affected. Oude-Engberink *et al*.^18^ confirmed that incubating human monocytes with USPIOs (1000 μg Fe/mL) results in efficient labeling detectable on MR images and does not affect cellular activity and activation markers such as cytokine production and cell migration. Furthermore, it has been proven that iron nanoparticles induce a significant increase in the production of ROS and subsequent microtubule remodeling^31^. Overproduction of ROS have been shown to be associated with various pathologic changes, such as cell structural damage, carcinogenesis, inflammation, radiation and reperfusion injury^32^. The uptake of iron oxide particles by cells is highly dependent on the cell types, cell size, surface charge, and coating of the particles. There is no single standard dosage that is appropriate for all cell types, thus we firstly explore the optimum concentration of USPIO for canine ADSCs labeling. As a cationic polymer, PLL can effectively boost the intracellular uptake of the nanoparticles through electrostatic interactions^15^. Compared with iron oxide alone, the iron nanoparticles and PLL mixture is more effective in achieving desired relaxation time^12^. Various concentrations of PLL have been used in the literature reports, from 0.375 μg/mL to 1.5 μg/mL^12^,^33^,^34^. Excessive PLL can form toxic aggregates and inhibit the nano-sized iron oxide incorporation in the cells, thus we chose a moderate concentration of PLL of 0.75 μg/mL. In this study, the zeta potential of 0.75 μg/mL PLL was detected as 5.33 mV, and it gradually dropped from 4.81 mV to 2.79 mV with mixing the increasing concentrations of USPIO from 5 μg Fe/mL to 100 μg Fe/mL. The positive lysine charges of the PLL/USPIO complexes balance the negative phosphate charges on the cell membrane, and further enhance the cell endocytosis^35^, which could contribute to the iron nanoparticles uptake and obviously reduce the dosage of USPIO.

**Figure 2:**
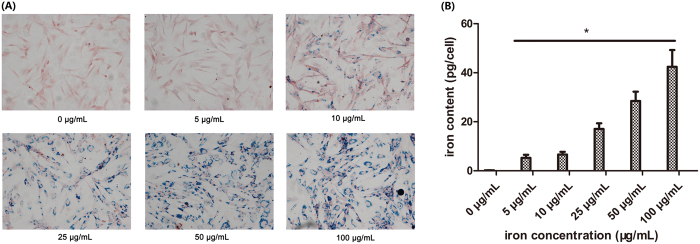
Prussian blue staining and iron quantification. (**A**): ADSCs were incubated for 12 h with varying iron concentrations of USPIO nanoparticles. Iron particles are stained blue (×200), scale bars: 100 μm. (**B**): quantitative analysis of iron oxides, the iron content per cell was detected by ICP-MS, the iron uptake increased with iron concentrations at a range of 5–100 μg/mL, *p < 0.05.

The iron content per cell increased with increasing incubation time, and reached saturation at 12 h of incubation (Supplemental Data 2). After incubation for 12 h with varying concentrations of USPIO nanoparticles, the labeling efficiency was detected by Prussian blue staining, as shown in Fig. 2A. The USPIO-labeled ADSCs exhibited an increasingly strong blue staining with the increasing concentrations of USPIO from 5–100 μg Fe/mL. Intracellular blue spots represent spread USPIO nanoparticles and mostly concentrated in the perinuclear region of the cytoplasm. ICP-MS in Fig. 2B showed the average iron content was 0.17 ± 0.036 pg/cell for 0 μg Fe/mL, 5.29 ± 1.225 pg/cell for 5 μg Fe/mL, 6.63 ± 1.117 pg/cell for 10 μg Fe/mL, 17.07 ± 2.325 pg/cell for 25 μg Fe/mL, 28.48 ± 3.806 pg/cell for 50 μg Fe/mL,42.42 ± 6.843 pg/cell for 100 μg Fe/mL. This indicated the iron content per cell is proportional to the iron concentrations in a range of 5–100 μg/mL (P < 0.05).

**Figure 3:**
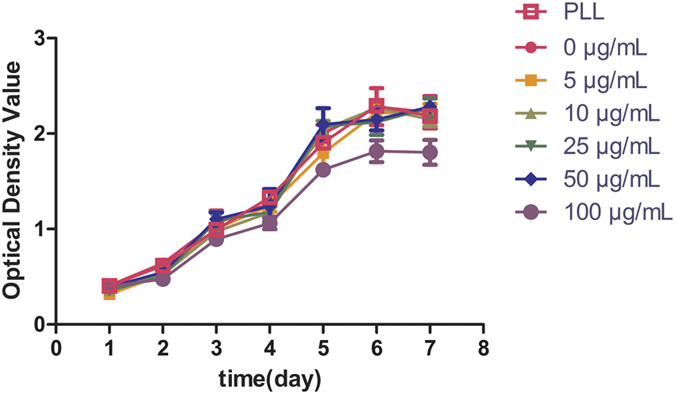
Cellular cytotoxicity assessment. ADSCs were co-cultured with USPIO nanoparticles at iron concentrations of 0–100 μg/ml, growth curves of USPIO-labeled ADSCs were obtained by CCK-8 assay. Compared with unlabeled cells, there was no cellular cytotoxicity of USPIO in the iron concentration range of 5–50 μg/ml and cells incubated with PLL alone, p > 0.05, while cell proliferation was inhibited at the iron concentration of 100 μg/ml, p < 0.05.

CCK-8 assay was used to detect the proliferation of USPIO-labeled cells and assess the cellular cytotoxicity of USPIO. The growth curves labeled cells with varying concentration of USPIO and unlabeled cells ware shown in Fig. 3. The data indicate, compared with unlabeled cells, no cellular cytotoxicity due to USPIO was found in the iron concentration range of 5–50 μg/ml and cells incubated with PLL alone, p > 0.05. The OD value of 100 μg Fe/mL labeled cells was significantly lower than unlabeled cells (p < 0.05). These results suggested that USPIO was not cytotoxic with good biocompatibility to ADSCs with a final iron concentration of less than 50 μg/ml.

**Figure 4:**
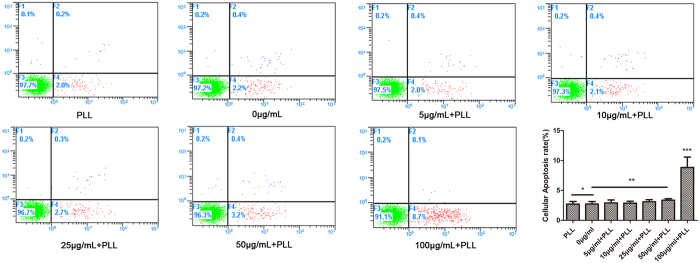
Cellular Apoptosis rate for varying iron concentration labeling. ADSCs were treated with USPIO nanoparticles at iron concentrations of 0–100 μg/ml and PLL alone. The cell apoptosis was demonstrated with flow cytometry. Compared with unlabeled cells (0 μg/ml), incubation of ADSCs with PLL alone did not affect the rate of apoptotic cells, *p = 0.892, and no significant difference was observed at an iron concentration of 5–50 μg/ml, **p > 0.05. However, USPIO could induce increased apoptosis of ADSCs at 100 μg Fe/ml concentrations, ***p < 0.01.

Furthermore, annexin V-FITC/PI double staining was performed to analyze the proportion of apoptotic cells in the varying concentration of USPIO-labeled and unlabeled cells. The results are shown in Fig. 4. The proportion of apoptotic cells in the USPIO-labeled cell samples was (2.9 ± 0.50)% for 5 μg/ml, (2.9 ± 0.32)% for 10 μg/ml, (3.1 ± 0.36)% for 25 μg/ml, (3.4 ± 0.25)% for 50 μg/ml, (8.8 ± 1.75)% for 100 μg/ml, whereas the proportion of apoptotic cells in the unlabeled cell (0 μg/ml) samples was (2.7 ± 0.42)% and cells incubated with PLL alone was (2.7 ± 0.46)%. Although the populations of apoptotic cells increased with concentration of USPIO, no significant difference was observed between the unlabeled and labeled cells at an iron concentration of 5–50 μg/ml, p > 0.05. Furthermore, the difference between unlabeled cells and cells incubated with PLL alone was not found to be statistically significant, p = 0.892. Compared with unlabeled cells, a statistically significant increase of apoptosis rate was apparent at 100 μg Fe/ml concentrations, p < 0.01. Thus, according to the labeling efficiency, cellular cytotoxicity and cellular apoptosis rate assessment, we chose to use USPIO at a Fe concentration of 50 μg/ml for a 12 h incubation, which can most efficiently mark cells and did not impair the cell survival, self-renewal, and proliferation capacity, and this concentration was screened to use for further experiments.

**Figure 5:**
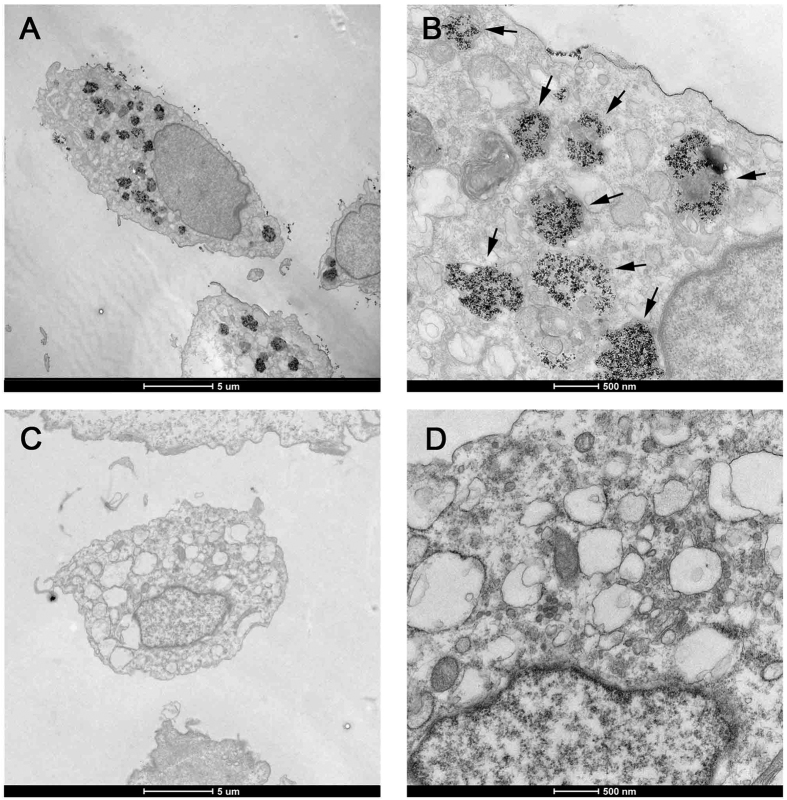
Transmission electron microscope for USPIO-labeled cells. Canine ADSCs were treated with USPIO nanoparticles at iron concentrations of 50 μg/ml, Transmission electron microscopy indicated, compared with unlabeled cells, the USPIO labeled cells can also keep its inherent structure and morphology, and labeled ADSCs had iron nano-particles (black granular material) in the cytoplasm, as indicated by arrowheads. (**A**): USPIO labeled ADSCs, ×1650, scale bar: 5 μm; (**B**): USPIO labeled ADSCs, ×11000, scale bar: 0.5 μm; (**C**): unlabeled ADSCs, ×1650, scale bar: 5 μm; (**D**): labeled ADSCs, ×11000, scale bar: 0.5 μm.

After 12 h incubation with an iron concentration of 50 μg Fe/ml, the uptake of USPIO particles into the cells was investigated using transmission electron microscopy. In Fig. 5A–D, similar to unlabeled ADSCs, the cellular structure and morphology of USPIO-labeled ADSCs were integral and clear, and the cell membrane did not appear to be damaged. USPIO was taken into cell through a membrane adsorption process and subsequent intracellular (non-nuclear) localization is found in endosomes^20^. Dark black spots indicate USPIO particles. A fraction of USPIO particles were found to be adhered to the cell plasma membrane and with no distribution in the nucleus, while most of them were incorporated into intracellular and aggregated in large lysosomes.

**Figure 6:**
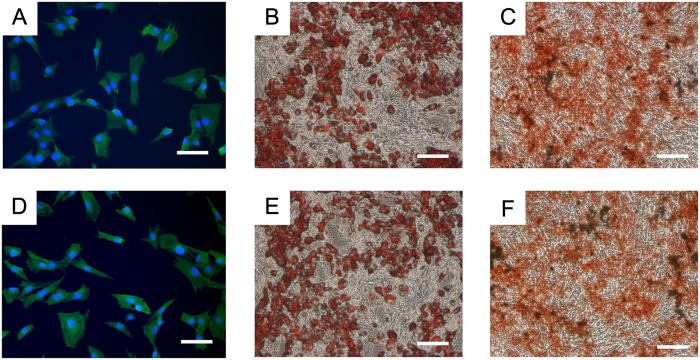
Myogenic, adipogenic and osteogenic differentiation of USPIO-labeled ADSCs after 3 weeks induction. Red-colored lipid vacuoles were detected by Oil Red staining. Calcium deposits were found in osteogenically differentiated ADSCs. Myoblast differentiation exhibited a significant upregulation of α-SMA expression. (**A**) α-SMA expression in USPIO-labeled ADSCs. (**B**) Oil red staining of USPIO-labeled ADSCs. (**C**) Alizarin red staining of USPIO-labeled ADSCs. (**D**) α-SMA expression in unlabeled ADSCs. (**E**) Oil red staining of unlabeled ADSCs. (**F**) Alizarin red staining of unlabeled ADSCs. Scale bars: 100 μm.

Several studies have suggested labeling stem cells with iron oxide in an appropriate concentration does not adversely affect the cell differentiation ability, including chondrogenic differentiation^36^, myogenic differentiation^37^, adipogenic differentiation^38^ and neural differentiation^39^. However, the biological effects of iron nanoparticles on osteogenic differentiation of stem cells remain controversial. Xiao *et al*.^40^ demonstrated that superparamagnetic iron oxide could promote osteogenic differentiation of rat ADSCs, while Fan *et al*.^41^ showed the osteogenic and adipogenic potential of ADSCs were inhibited after SPIO labeling. One possible reason is that SPIOs can influence cell mobilization, involve the activation of relevant signaling pathway and further affect osteogenic differentiation ability^42^. High iron nanoparticle loads could have negative effects on cell viability and differentiation, thus it is important to choose the appropriate concentration. In our study, the USPIO-labeled ADSCs maintained a good differentiation potential and no significant difference was observed in the myogenic (Fig. 6A vs Fig. 6D), osteogenic (Fig. 6B vs Fig. 6E) and adipogenic (Fig. 6C vs Fig. 6F) differentiation ability *in vitro* between the USPIO-labeled and unlabeled groups (Fig. 6).

**Figure 7:**
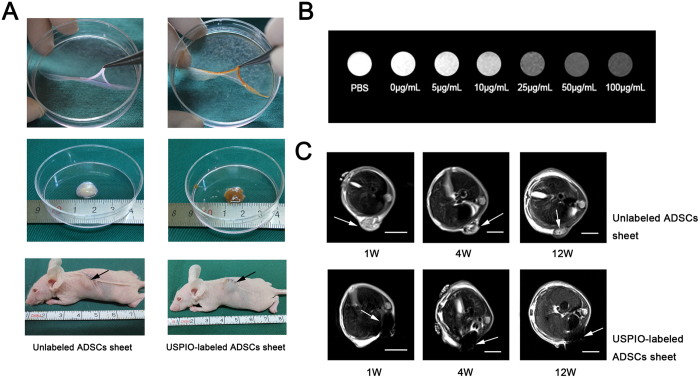
Tracking of USPIO-labeled ADSCs sheet by MRI. (**A**): Unlabeled (control) and 50 μg Fe/mL labeled ADSCs sheets were gathered to cell sheet “pellets” with cell scraper, about diameter 1 cm and length 1 cm, and then subcutaneously transplanted in the back of nude mice. (**B**): T2-weighted MR images of USPIO-labeled ADSCs *in vitro*. 2 × 10^5^ ADSCs were treated with USPIO at the iron concentrations of 0–100 μg/ml and PBS was taken as control, the signal intensity is proportional to the concentrations of USPIO, the gray difference can be easily identified by naked eye at the iron concentration of greater than or equal to 25 μg/ml. (**C**): MRIs were examined at 1 week, 4 weeks, and 12 weeks after USPIO-labeled ADSCs sheet transplantation, white arrows represent the area of the USPIO-labeled and unlabeled ADSCs sheet, scale bar: 1 cm.

To test the detectability in MRI, 2 × 10^5^ varying concentrations of USPIO-labeled and unlabeled ADSCs were placed in 1.5 mL tube (Fig. 7A) and were detected as strongly hypointense areas on T2-weighted images (Fig. 7B). A good correlation between the image grey-scale value and the concentrations of USPIO in the MR images was observed. Lighter gray represents lower iron concentrations and darker gray represents higher iron concentrations. There are significant differences in the gray value among varying concentrations of USPIO. Moreover, the gray difference can be easily identified by the naked eye at concentrations greater than or equal to 25 μg Fe/ml. These results indicated that MRI can efficiently detect USPIO-labeled ADSCs and that the intensity of the signal is proportional to the concentrations of USPIO.

Vitamin C was added to culture medium to accelerate native ECM secretion and generate a three dimensional tissue-like and contiguous cell sheets^43^. Various types of ECM and membrane proteins penetrate into the cell sheets and play a role as adhesive agents to limit cell distribution and migration. Thus cell sheets are intact whole before and after transplantation. Furthermore, MR imaging is applicable to continuously track the migration and location of transplanted cells, even for a small number of labeled cells (5 × 10^3^ to 1 × 10^4^)^44^ or single cell images^45^. For cell sheets, the cell number is very large. The number of initial seeding cells is 1.05 × 10^6^ cells per sheet in our experiments. The characteristics of large quantities of cells are favorable for detection of USPIO-labeled cell sheet by MRI imaging. For USPIO, the effect of MR contrast agents is not observed directly on the image, but the change of the relaxation rate of water protons is detectable by MRI^46^. Thus, iron oxide nanoparticles are seen as hypointensity or negative contrast on T2-weighted and T2*-weighted images and often referred to as “negative” contrast agents. *In vitro* MR imaging showed, compared with unlabeled cells or PBS, a visible hypointense signal can be provided at the concentrations of 25 μg Fe/mL.

For *in vivo* MR imaging, the USPIO-labeled cell sheet “pellets” implants were visible as a hypointense area at the transplantation site and three-dimensional images could be further provided. Several studies have suggested the use of iron oxide nanoparticles *via* cell labeling as an effective MR contrast for detecting grafted cells and tissue over long times in animal experiments. Bulte *et al*.^20^ showed SPIO-labeled oligodendroglial progenitors can be readily detected *in vivo* for 6 weeks after transplantation. Neri *et al*.^44^ proved that low numbers of viable SPIO-labeled human neural precursor cells (5 × 10^3^ to 1 × 10^4^) could be efficiently detected in the adult murine brain after transplantation, and it could be tracked for at least 1 month *in vivo*. Furthermore, Guzman *et al*.^47^ detected the targeted migration and distribution of SPIO-labeled clusters of cells up to 18 weeks. For monitoring of cell sheet transplantation, we further localize USPIO-labeled cell sheet by MRI after transplantation in the subcutaneous area of nude mice for at least 12 weeks. At 1 week, 4 weeks and 12 weeks after transplantation into the subcutaneous area of nude mice, we explored whether USPIO-labeled ADSCs sheet “pellets” could be localized by MRI, as shown in Fig. 7C. One week after implantation, regions of hypointense signal in T2 images were found in the transplantation sites of 50 μg Fe/ml USPIO-labeled ADSCs sheet “pellets” and no MRI signals were detected when similar volume unlabeled ADSCs sheet “pellets” were transplanted. The signal was visualized up to 4 weeks and even 12 weeks after transplantation. Thus, our result showed USPIO-labeled ADSCs sheets can be easily and clearly detected *in vivo* and has persisted for at least 12 weeks. However, compared with 1 week after implantation, grey-scale value and area of hypointense signal nearly remain the same at 4 weeks after implantation and deceased at 12 weeks.

**Figure 8:**
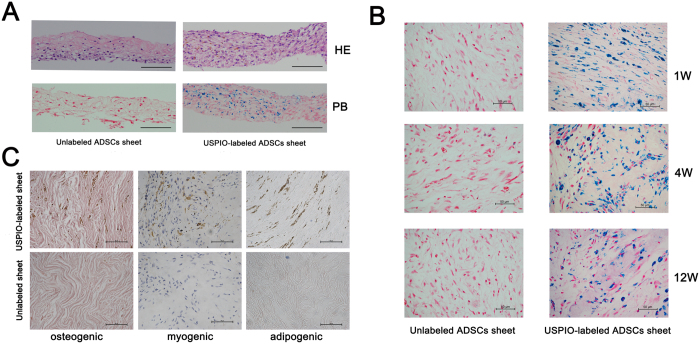
Histological analysis and differentiation of USPIO-labeled ADSCs sheet *in vivo*. 50 μg Fe/ml USPIO-labeled and unlabeled ADSCs continuously culture and form cell sheet with the stimulation of vitamin C. (**A**): Histological analysis of USPIO-labeled cell sheet *in vitro*, HE represent HE stained, PB represent Prussian blue staining, scale bar: 100 μm. (**B**) Unlabeled (control) and 50 μg Fe/mL labeled ADSCs sheet were subcutaneously transplanted in the back of nude mice. Prussian blue staining for iron after 1, 4, and 12 weeks after transplantation, the blue-stained iron particles in the cytoplasm decrease gradually from week 1 to week 12, scale bar: 50 μm. (**C**) After 12 weeks of implanting, no spontaneous osteogenic, adipogenic and myogenic differentiation was observed in both USPIO-labeled and unlabeled ADSCs sheet, scale bar: 50 μm.

Histological analysis in Fig. 8A revealed that USPIO particles were a granular brown substance which was located mainly in the cytoplasm and there was no difference in cell morphology between labeled and unlabeled cells in the HE stained section. Prussian Blue staining revealed positive stained granules exclusively in the transplants of USPIO-labeled cells and presented an even distribution within the cell sheet. However, the blue-stained iron particles in the cytoplasm decrease gradually from week 1 to week 12 when these were transplanted into subcutaneous area (Fig. 8B). Signal intensity was well correlated with iron accumulation. In our study, over 12 weeks the signal intensity of USPIO-labeled cell sheet reduced gradually with time, which was also proven by Prussian Blue staining. We analyzed the possible cause of the changes of signal intensity, including: 1) A fraction of the USPIO-labeled cells gradually migrating out of the cell sheet. 2) The iron particles in the magnetic labeled cells can be passed to offspring cells, and the concentration of iron particles can be diluted and decrease during cell division. 3) Iron oxide loading has been shown proved to increase activity of lysosomal cathepsin D, and the latter induces the cellular iron to gradually degrade in lysosomes^48^. Although most of the iron particles remains intact in the endosome^49^, some amount of the nano-sized iron oxide can be released from the labeled cell by exocytosis with time.

In addition, the osteogenic, adipogenic and myogenic differentiation were measured to evaluate the cell differentiation *in vivo* (Fig. 8C). The result showed, apart from brown iron particle deposited within USPIO-labeled cell sheets, no spontaneous cell differentiation towards bone, fat and muscle was observed in both USPIO-labeled and unlabeled ADSCs sheets. Our study certified the feasibility of tracking ADSCs sheet with USPIO *in vivo* for a long time (12 weeks), however, due to the xenoplastic transplantation of ADSCs sheet, it is hard to detect the cell migration in this study, and further studies may focus on the cell sheet migration towards deep anatomical sites in homoplastic transplantation.

## Conclusion

In summary, the ADSCs sheet can be effectively labeled with 50 μg Fe/ml USPIO and 0.75 μg/mL PLL. The particles did not impair cell survival, self-renewal, and proliferation capacity. USPIO might be feasible for *in vivo* labeling of the ADSCs sheets using the optimal concentration of 50 μg Fe/ml. This level of concentration did not influence the cells’ activity nor the cell apoptosis rate. MRI scanning on T2WI sequences can effectively track the USPIO nanoparticles labeled ADSCs *in vivo* for at least 12 weeks. MRI can be used to continuously track the ADSCs sheet labeled with USPIO transplanted into nude mice and provide a non-invasive method to understand the fate of the labeled cells in the transplanted areas.

## Additional Information

**How to cite this article:** Zhou, S. *et al*. Labeling adipose derived stem cell sheet by ultrasmall super-paramagnetic Fe_3_O_4_ nanoparticles and magnetic resonance tracking *in vivo. Sci. Rep.* **7**, 42793; doi: 10.1038/srep42793 (2017).

**Publisher's note:** Springer Nature remains neutral with regard to jurisdictional claims in published maps and institutional affiliations.

## Supplementary Material

Supporting Information
