# Supplementary material for: Labeling adipose derived stem cell sheet by ultrasmall super-paramagnetic Fe3O4 nanoparticles and magnetic resonance tracking in vivo
Source: Sci Rep. 2017 Feb 21;7:42793. doi: 10.1038/srep42793 (PMC5318892; doi:10.1038/srep42793)
Supplement: Supporting Information [file srep42793-s1.doc]

Labeling adipose derived stem cell sheet by ultrasmall super-paramagnetic Fe_3_O_4_ nanoparticles and magnetic resonance tracking *in vivo*

Shukui Zhou^1,†^, Ting Yin^2,†^, Qingsong Zou^1^, Kaile Zhang^1^, Guo Gao^2,*^, Joseph G. Shapter^3^, Peng Huang^2^, Qiang Fu^1,*^

^1^ Department of Urology, Affiliated Sixth People’s Hospital, Shanghai Jiao Tong University, Shanghai, China.

^2^ Institute of Nano Biomedicine and Engineering, Shanghai Engineering Research Center for Intelligent Diagnosis and Treatment Instrument, Department of Instrument Science and Technology, School of Electronic Information and Electrical Engineering, Shanghai Jiao Tong University, Shanghai, China.

^3^ School of Chemical and Physical Sciences, Flinders University, Bedford Park, Adelaide 5042, Australia.

†These authors contributed equally.

*Corresponding author, Qiang Fu, Department of Urology, Affiliated Sixth People’s Hospital, Shanghai Jiao Tong University, Shanghai, China. ZIP: 200233. Fax: 021-63868708, E-mail address: [jamesqfu@aliyun.com](mailto:jamesqfu@aliyun.com).

Guo Gao, E-mail addresses: [guogao@sjtu.edu.cn](mailto:guogao@sjtu.edu.cn).

Supporting Information

Supplementary Data 1

The identification of ADSCs

**Methods**

The ADSCs were seeded into 6-well tissue microplates. When the cells reached 80% confluence, the hematopoietic markers CD45, CD34 and the stem cell markers CD90 E CD105 were evaluated by flow cytometry. The cells were fixed with 4% paraformaldehyde (10 min) and then permeabilized with 0.1% PBS-Tween for 20 minutes. ADSCs were washed twice with PBS at each staining with a combination of canine antibodies attached to fluorochromes, specific for CD34 PE (eBioscience, San Diego, CA, USA), CD44 FITC (eBioscience), CD45 FITC (eBioscience), CD90 PE (eBioscience), CD105 FITC (Abcam, Cambridge, MA, USA). Cells were incubated with the antibodies for at least 30 min at 22°C in the dark. Normal canine ADSCs were stained with Mouse IgG1 kappa Isotype control PE for CD34 (eBioscience), Rat IgG2a kappa Isotype control FITC for CD44 (eBioscience), Rat IgG2b Kappa Isotype Control FITC for CD45 (eBioscience), Rat IgG2b kappa Isotype Control PE for CD90 (eBioscience) and Mouse Monoclonal IgG2b FITC for CD105 (Abcom). An unlabeled sample was also used as a blank control. After washing twice with PBS, the samples were analyzed by flow cytometry.

**Results**

The primary cultured ADSCs adhered and quickly proliferated in vitro. ADSCs showed a central spiral distribution and a long fusiform shape with single nuclei. After inoculation, ADSCs grew for 24 hours and started to proliferate after 72 hours. The cellular fusion rate was between 80%-90% on day 7. Flow cytometry analysis indicated, the primary cultured ADSCs in our study were negative for hematopoietic markers CD34 and CD45, and were strongly positive for MSC-related markers CD44, CD90 and CD105, which confirmed the stem cell origin of ADSCs.


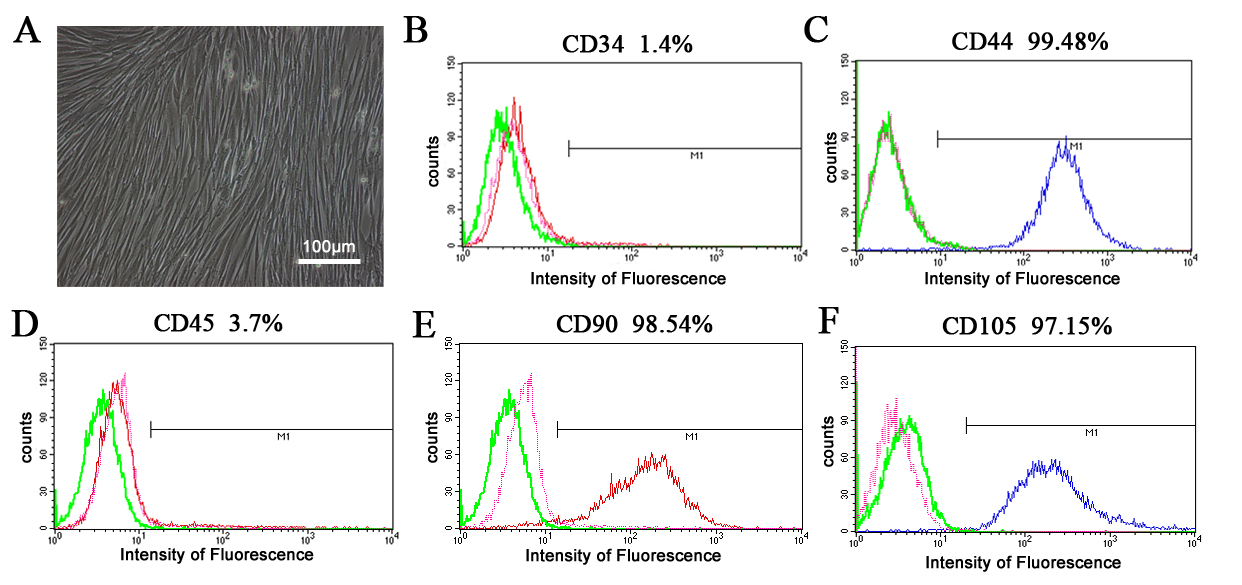


Fig. S1 Isolation and identification of canine ADSCs. (A) The primary cultured ADSCs were isolated from fat tissue of beagle dogs, cultured in 100mm cell culture dish (×100). Scale bars of 100μm. (B-F) The expression level of ADSCs surface antigens was determined by flow cytometry, including CD45, CD90 and CD105. The ADAS cells rarely express the hematopoietic markers CD45, but consistently express CD90 and CD105. The experiment was repeated three times, and data represent the mean values of 3 individual experiments.

Supplementary Data 2

Methods

In order to determine the optimal incubation time, cells were labeled with 25 μg Fe/mL USPIO (a moderate concentration) for different times (1, 3, 6, 12 and 24 h), then the USPIO-labeled cells were trypsinized, detached, counted and resuspended in a 37% HCl solution. The iron content per cell was obtained by ICP-MS. Three replicates at each concentration were measured for statistical analysis.

**Results**

The uptake of USPIO was time-dependent. USPIOs were rapidly internalized into the cells within the first 1 h, and the iron uptake increases with time, reaching 8.32 ± 0.811 pg/cell, 10.74 ± 0.945 pg/cell, 12.48 ± 1.359 pg/cell, 17.79 ± 2.041 pg/cell, 19.02 ± 1.983 pg/cell at 1, 3, 6, 12, 24 h, respectively. Though a higher uptake could be observed at 24 h compared to 12 h, the two results were not statistically significantly different, which showed the saturated uptake was reached after 12 h of incubation.


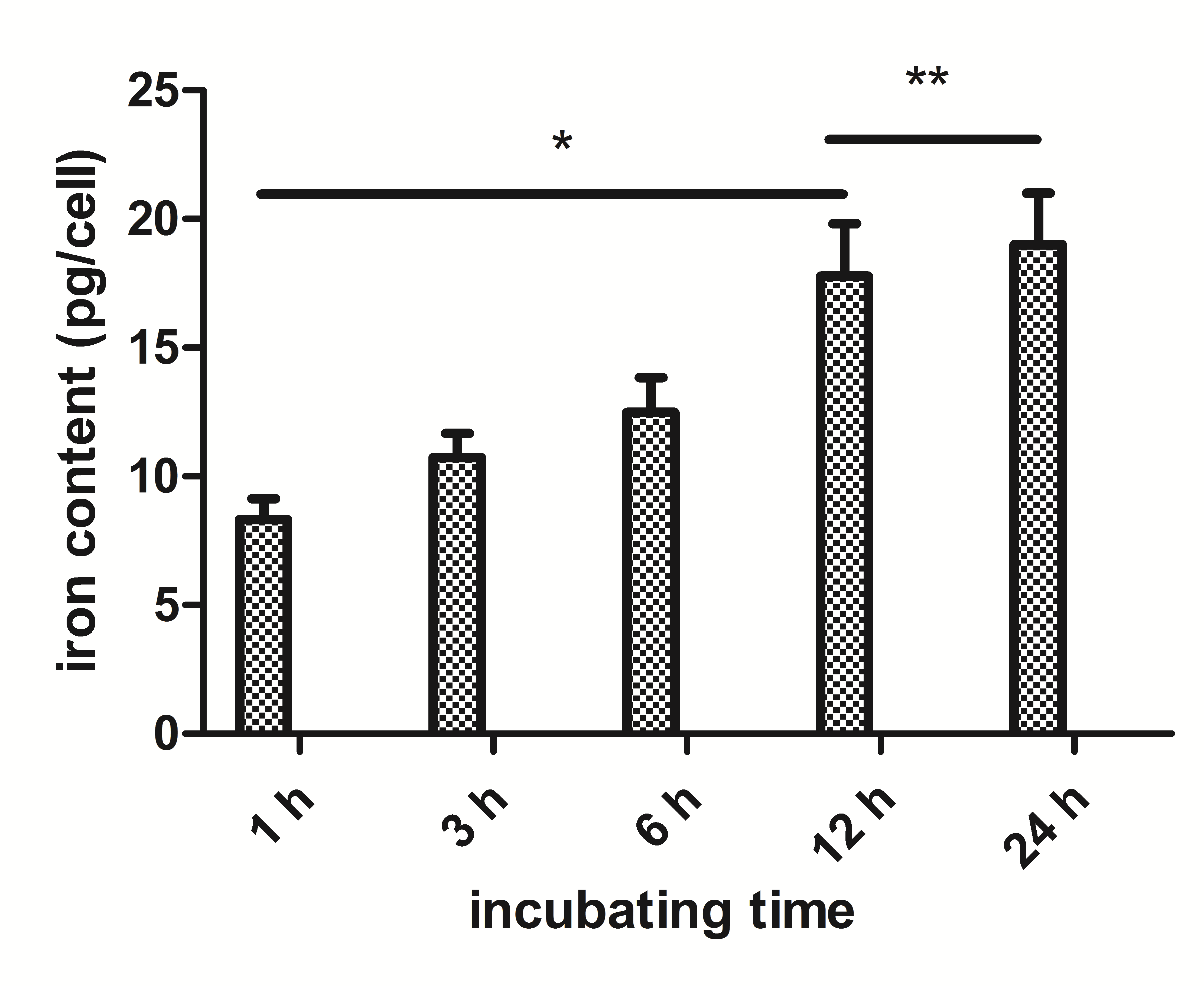


Fig. S2 iron quantification at different incubation time periods (1, 3, 6, 12 and 24 h). The iron

content increased with incubation time prolonged, and reached saturation at 12 h of incubation. * p<0.05, **p>0.05.
